# Mathematically optimized cryoprotectant equilibration procedures for cryopreservation of human oocytes

**DOI:** 10.1186/1742-4682-11-13

**Published:** 2014-03-20

**Authors:** Allyson Fry Davidson, James D Benson, Adam Z Higgins

**Affiliations:** 1 School of Chemical, Biological and Environmental Engineering, Oregon State University, 102 Gleeson Hall, Corvallis, Oregon 97331-2702, USA; 2 Department of Mathematical Sciences, Northern Illinois University, DeKalb, IL 60115-288, USA

**Keywords:** Optimization, Toxicity, Vitrification, Cell membrane transport, Permeability

## Abstract

**Background:**

Simple and effective cryopreservation of human oocytes would have an enormous impact on the financial and ethical constraints of human assisted reproduction. Recently, studies have demonstrated the potential for cryopreservation in an ice-free glassy state by equilibrating oocytes with high concentrations of cryoprotectants (CPAs) and rapidly cooling to liquid nitrogen temperatures. A major difficulty with this approach is that the high concentrations required for the avoidance of crystal formation (vitrification) also increase the risk of osmotic and toxic damage. We recently described a mathematical optimization approach for designing CPA equilibration procedures that avoid osmotic damage and minimize toxicity, and we presented optimized procedures for human oocytes involving continuous changes in solution composition.

**Methods:**

Here we adapt and refine our previous algorithm to predict piecewise-constant changes in extracellular solution concentrations in order to make the predicted procedures easier to implement. Importantly, we investigate the effects of using alternate equilibration endpoints on predicted protocol toxicity. Finally, we compare the resulting procedures to previously described experimental methods, as well as mathematically optimized procedures involving continuous changes in solution composition.

**Results:**

For equilibration with CPA, our algorithm predicts an optimal first step consisting of exposure to a solution containing only water and CPA. This is predicted to cause the cells to initially shrink and then swell to the maximum cell volume limit. To reach the target intracellular CPA concentration, the cells are then induced to shrink to the minimum cell volume limit by exposure to a high CPA concentration. For post-thaw equilibration to remove CPA, the optimal procedures involve exposure to CPA-free solutions that are predicted to cause swelling to the maximum volume limit. The toxicity associated with these procedures is predicted to be much less than that of conventional procedures and comparable to that of the corresponding procedures with continuous changes in solution composition.

**Conclusions:**

The piecewise-constant procedures described in this study are experimentally facile and are predicted to be less toxic than conventional procedures for human oocyte cryopreservation. Moreover, the mathematical optimization approach described here will facilitate the design of cryopreservation procedures for other cell types.

## Introduction

Cryopreservation theoretically allows nearly indefinite storage of viable biological material [1]. Conventional cryopreservation techniques are usually thought of as slow-cooling methods (~1°C/min) that utilize relatively low (1 to 2 mol/L) concentrations of cryoprotectants (CPAs) such as glycerol, ethylene glycol, or dimethyl sulfoxide. Although these conventional techniques are sufficient for many cell types, this approach is less successful for cells that have a reduced tolerance to sub-physiologic temperatures (e.g. oocytes [2,3]) or are easily damaged by extracellular ice formation (e.g., three dimensional tissues [4,5]). For these sensitive cell types, an alternative cryopreservation technique widely known as vitrification may be used that preserves cells in a glassy state devoid of ice crystals.

In order to completely avoid the liquid to crystal phase transition, these vitrification techniques require combinations of very high cooling and warming rates (typically >>100°C/min) with cryopreservation solutions that contain very high concentrations of CPA (typically > 5 mol/L). In addition to avoiding damage associated with ice formation, vitrification techniques are appealing because they require much less precise cooling rates compared to conventional methods, and as such can be implemented without costly or complicated controlled rate freezing devices.

However, there is a high cost associated with these techniques: the equilibration of cells with and from high CPA concentrations (CPA addition and removal, respectively) dramatically increases the risk of damage due to osmotically driven cell volume changes and CPA induced cytotoxicity. Volumetric damage can be caused by rapid exposure to anisosmotic media, during which the differential permeability of water and CPA drives a biphasic volume response. This damage occurs when the cell either rapidly loses and then slowly regains its intracellular water in traditional CPA addition schemes, or vice versa with traditional removal schemes. These responses, if large enough, may drive the cell beyond critical volumes known as osmotic tolerance limits, outside of which irreversible cell damage occurs [6,7]. Additionally, high CPA concentrations also increase the risk of cell damage or death due to chemical toxicity; it has been claimed that preventing toxicity is the biggest challenge in achieving successful vitrification [8].

Rational design approaches combine mathematical models and cell biophysical parameters to predict optimized CPA addition and removal procedures. Because the damage due to extending cell volumes beyond osmotic tolerance limits is relatively well understood, the most common rational design method has been to use membrane transport equations and osmotic tolerance limits to predict multi-step procedures that prevent osmotic damage [9-11]. With an argument that cytotoxicity due to CPA exposure is time-sensitive, rational design strategies have also been extended to reduce toxic damage by minimizing the duration of the CPA addition and removal procedures while still maintaining cell volumes between osmotic tolerance limits [12,13].

While CPA cytotoxicity is time sensitive, it is also concentration sensitive [8,14,15]. Therefore, in order to account for this time and concentration dependence, we recently described mathematical methods that predict optimal procedures based on the minimization of a toxicity cost function, a term that describes the accrual of toxic damage [16]. However, our previous mathematical algorithm predicted procedures with continuous concentration changes for CPA and non-permeating solutes. These procedures are difficult to implement and would require specialized fluidic systems and computerized control. Moreover, because most previous rationally designed procedures used an isosmotic volume as the final state for CPA addition and removal [9,10], our previous study used an isosmotic volume to define the target final cellular state. This final state may be less optimal than one where the cell is dehydrated to its osmotic tolerance limit at the end of CPA loading. In fact there has been discussion in the literature about the advantages of cooling in a pre-dehydrated state (see, e.g., [17]).

In the current study, we describe adaptations to our previous algorithm in order to make the predicted procedures easier to implement. The minimization of a toxicity cost function remains the basis of our algorithm. However, instead of predicting procedures with continuous concentration changes, the new algorithm predicts multi-step procedures with piecewise constant changes in the CPA and non-permeating solute concentrations. Also, rather than specifying an isotonic final cell volume, the new algorithm uses the intracellular CPA concentration to define the target final state, which allows exploration of alternate final cell volumes. We predict procedures for the addition and removal of vitrification solutions for human oocytes; a valuable, clinically relevant, and challenging to cryopreserve cell type. Our results demonstrate the potential to significantly reduce the toxicity of vitrification procedures with an experimentally and clinically facile CPA equilibration protocol.

## Methods

Our approach for optimizing CPA addition and removal procedures involves minimization of a toxicity cost function subject to cell membrane transport equations and cell volume state constraints. To achieve this minimization, we used cell membrane transport predictions to both evaluate the state dependent toxicity cost function and to ensure that cell volumes did not violate the osmotic tolerance constraints. To model the cellular state, we used the nondimensional form of the two parameter membrane transport model [16,18]:

(1) $$\begin{array}{l} \frac{\mathrm{dw}}{\mathrm{d\tau}}=-m_{1}-m_{2}+\frac{1+s}{w},\\ \frac{\mathrm{ds}}{\mathrm{d\tau}}=b\left(m_{2}-\frac{s}{w}\right), \end{array}$$

where *w* is the intracellular water volume normalized to the water volume under isotonic conditions, *s* is the moles of intracellular CPA normalized to the moles of intracellular solute under isotonic conditions, *τ* is a dimensionless temporal variable, *b* is a dimensionless relative permeability constant, and *m*_1_ and *m*_2_ are the extracellular concentrations (in molal units) of non-permeating solute and CPA, respectively, normalized to the isotonic solute concentration (0.3 Osm/kg).

For human oocytes exposed to ethylene glycol (EG) at 22°C, published membrane permeability values yield a relative permeability constant of *b* = 1.62 [19]. These permeability values also result in a dimensional time (in minutes) that is 4.33 times larger than the nondimensional time. Osmotic tolerance data for human oocytes [7,20] were used to define constraints on the cell volume, yielding

(2) 0.47≤w+γs≤1.67,

where *γ* is the product of the isotonic solute concentration and the partial molar volume of CPA. In the case of EG, *γ* = 0.0168.

As in our previous study, we used a toxicity cost function based on published toxicity data for exposure of cartilage [14] and fibroblasts [15] to dimethyl sulfoxide. The cost function can be expressed as

(3) $$J_{\alpha }=\int_{0}^{\tau^{f}}\frac{s^{\alpha }}{w^{\alpha }}\;\mathrm{d\tau},$$

where *α* = 1.6 is a constant describing the concentration dependence of the toxicity rate, and *τ*^f^ is the total duration of the procedure [16].

In the previous implementation of our optimization approach [16], we defined the goal state (i.e., the desired final state at the end of the procedure) as a specific set of state variable values, *w*^f^ and *s*^f^. In particular, for addition of EG, we used the values *w*^f^ = 0.67 and *s*^f^ = 19.9, which correspond to an intracellular EG concentration of 6 mol/L (*s*^f^/*w*^f^ = 30) and a cell volume equivalent to the isotonic cell volume (*w*^f^ + *γs*^f^ = 1). To ensure that the optimization algorithm terminated at the goal state, we minimized a cost function equal to

(4) $$J_{\alpha ,\epsilon }=J_{\alpha }+J_{\epsilon },$$

where *J*_ϵ_ is a cost associated with the proximity of the final state to the goal state, and is defined as

(5) $$J_{\epsilon }=\frac{1}{\epsilon }\left({\left(w\left(\tau^{f}\right)-w^{f}\right)}^{2}+{\left(s\left(\tau^{f}\right)-s^{f}\right)}^{2}\right).$$

In the present study, we investigated an alternative definition of the goal state. Rather than uniquely specifying the values of both *w*^f^ and *s*^f^, we chose a specific intracellular EG concentration as the goal state. This goal state definition is consistent with the purpose of CPA loading for vitrification methods: to achieve an intracellular CPA concentration that enables vitrification of the intracellular solution at practicable cooling and warming rates. For example, if we wish to achieve an intracellular EG concentration of 6 mol/L, then our goal state is *s*^f^/*w*^f^ = 30, defining a line in the *s*, *w* state space. For CPA removal, we define the goal state as *s*^f^/*w*^f^ = 0, again not limiting our goal state to an isotonic volume. With the goal state defined in this way, we redefined the proximity cost as

(6) $$J_{\epsilon }=\frac{1}{\epsilon }{\left(\frac{s\left(\tau^{f}\right)}{w\left(\tau^{f}\right)}-\frac{s^{f}}{w^{f}}\right)}^{2}.$$

We used *ϵ* = 10^−3^ for CPA addition and *ϵ* = 10^−1^ for CPA removal, which was found to result in convergence near the goal state.

In order to identify optimal CPA addition and removal procedures it is first necessary to parameterize the procedural details. We assumed a constant temperature and only considered the solute concentrations *m*_1_(*τ*) and *m*_2_(*τ*) in the optimization scheme. In our previous study, we parameterized *m*_1_(*τ*) and *m*_2_(*τ*) using a piecewise linear approach [16]. The temporal domain between *τ* = 0 and *τ*^f^ was divided into 49 equally spaced segments and the concentrations *m*_1_ and *m*_2_ were assumed to vary linearly with time in each segment. This corresponds with 50 parameters for *m*_1_, 50 parameters for *m*_2_ and one additional temporal parameter *τ*^f^, resulting in a total of 101 parameters to be optimized.

One of the goals of the present study was to modify the optimization approach to yield procedures that are easier to implement experimentally. Thus we examined procedures consisting of piecewise constant concentration profiles for *m*_1_(*τ*) and *m*_2_(*τ*). We considered both two-step and three-step procedures—procedures with either two or three step-changes in the extracellular concentration. In the case of two-step procedures, the parameters to be optimized consist of the duration of the first step, the concentrations *m*_1_ and *m*_2_ in the first step, the duration of second step and the concentrations *m*_1_ and *m*_2_ in the second step, resulting in a total of 6 parameters. A total of 9 parameters are required for parameterization of three-step procedures. Unless otherwise noted, the concentration parameters to be optimized were bounded between a lower limit of *m* = 0 and an upper limit of *m* = 80. This corresponds with a maximal EG concentration of 60% w/w, or about 10.3 mol/L.

A convenient outcome of assuming piecewise constant concentration profiles for *m*_1_(*τ*) and *m*_2_(*τ*) is that an analytical solution to system (1) is available when *m*_1_ and *m*_2_ are constant [21]. The use of the analytical solution dramatically improves the convergence speed and the stability of the calculation in comparison to the use of numerical methods for solving the differential equations. As described in Benson et al. [21], the basic approach for finding the analytical solution is to define a grouped variable that includes both the time and the cell water volume in order to convert the membrane transport model into a set of linear differential equations that can be solved using standard methods (see, e.g., [22]). In terms of the nondimensional variables in system (1), the new time-like variable *x* is defined by the relationship

(7) $$\mathrm{dx}=\frac{1}{w}\mathrm{d\tau}.$$

The time variable transformation alters the cost function (Eq. 3) that now may be rewritten equivalently in terms of *x*,

(8) $$J_{\alpha }=\int_{0}^{x^{f}}\frac{s^{\alpha }}{w^{\alpha -1}}\;\mathrm{dx},$$

allowing the calculation and optimization to occur completely in the time transform space with the attendant exact solutions. The analytical solutions for *w* and *s* in terms of the variable *x* are provided in the Appendix.

To mathematically optimize piecewise constant procedures, the built-in constrained minimizer “fmincon” was used in MATLAB (MathWorks, Inc., Natick, MA) to implement the interior point algorithm [23-25]. This algorithm was used to minimize the value of the cost function (Eq. 4) subject to the constraints in Eq. 2, and a grid search approach was used with a wide range of initial parameter guesses to increase the potential for finding a global minimum. In practice, we found that several parameter combinations yielded nearly identical cost function values, an observation that is consistent with previous attempts to optimize piecewise constant CPA addition and removal procedures [12]. Consequently the “optimal” procedures reported here probably do not represent true global optimums, but rather procedures in the vicinity of the global optimum. Finally, to compare our new approach to non-piecewise constant controls, we solved the continuous control problem as before [16] but without the *w*^f^ + *γs*^f^ = 1 condition; i.e., we simply replaced the previous end point penalty cost function *J*_ϵ_ (Eq. 5) with its new expression (Eq. 6).

## Results

**Figure 1:**
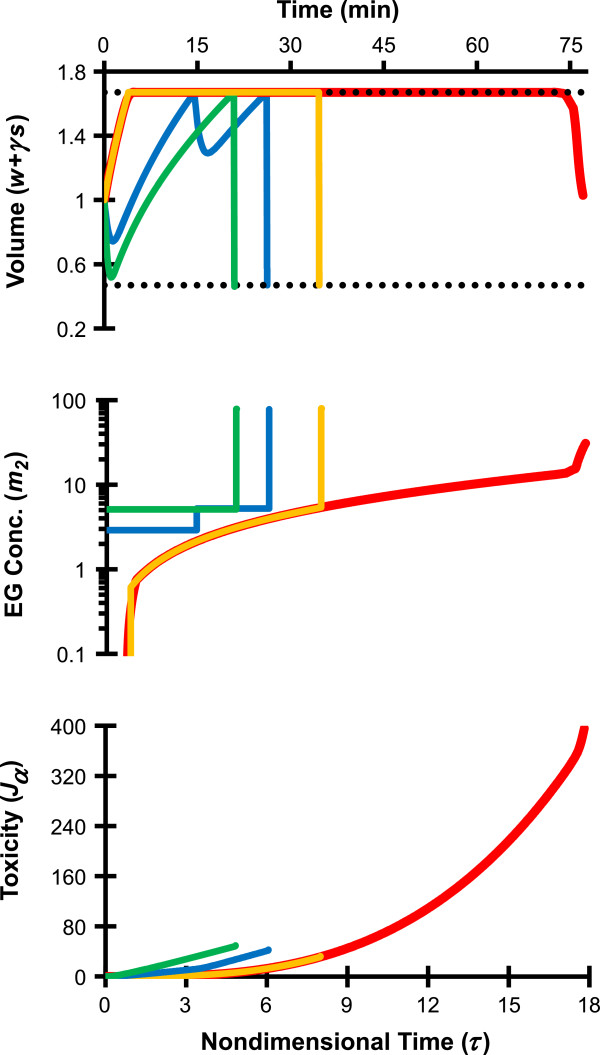
**Comparison of mathematically optimized protocols for equilibration of human oocytes with EG.** All of the procedures terminated at a goal state with *s*^f^/*w*^f^ = 30, which is equivalent to an intracellular EG molality of 9 Osm/kg, or a molar concentration of about 6 mol/L. The red line shows results from our previous study [16], which involved piecewise linear parameterization of *m*_1_(*τ*) and *m*_2_(*τ*) and a goal state fixed at the isotonic cell volume. The orange line shows results for the same piecewise linear parameterization of *m*_1_(*τ*) and *m*_2_(*τ*), but with a goal state that was not fixed at a specified final volume. The green and blue lines show two-step and three-step piecewise constant procedures, which also had goal states that were not fixed at a specific final volume. The horizontal dotted lines in the top figure show the osmotic tolerance limits. Note that the nondimensional EG concentration *m*_2_ can be converted to molal units by multiplying by 0.3 Osm/kg.

To allow storage of oocytes in an ice-free glassy state it is first necessary to equilibrate the cells in a sufficiently concentrated CPA solution so that the sample vitrifies during cooling and does not devitrify (crystallize) during warming. We initially considered 6 mol/L EG to be a “vitrifiable” concentration, and used an intracellular EG concentration of 6 mol/L as the target state at the end of CPA loading. Figure 1 compares two different strategies for defining the target final state (i.e., the goal state) in the optimization algorithm. The first strategy was that of our previous study where the goal state satisfied the following two conditions: (1) an intracellular EG concentration of 6 mol/L (*s*^f^/*w*^f^ = 30), and (2) a final cell volume equal to the isotonic cell volume (*w*^f^ + *γs*^f^ = 1). For the second strategy, the goal state still consisted of an intracellular EG concentration of 6 mol/L (*s*^f^/*w*^f^ = 30), but the final cell volume was not specified. To compare these goal state definitions, EG loading procedures were designed using a piecewise linear parameterization of the extracellular concentrations *m*_1_ and *m*_2_ (i.e., the concentrations were allowed to vary continuously with time); the resulting procedures are shown with red and orange lines, respectively. In both cases, the mathematically optimized procedures called for a non-permeating solute concentration *m*_1_ that was zero throughout the EG addition process. Thus, all EG loading solutions contained only EG and water. Also, for both approaches the cells were initially induced to swell to the maximum cell volume limit (as defined in Eq. 2) by exposure to hypotonic solution. In this swelling phase of the procedure, very little EG was loaded into the cells, because the extracellular solution contained a very low EG concentration. Once the upper volume limit was reached, the EG concentration was increased and maintained near osmotic equilibrium at a concentration that resulted in a volumetric influx of EG that was exactly balanced by efflux of water. The resulting constant-volume period can be thought of as the EG loading phase of the procedure. At the end of the EG loading phase, the extracellular EG concentration was abruptly increased, causing the cells to shrink rapidly due to water efflux. This shrinkage concentrated the intracellular EG that had been introduced during the loading phase. When the goal state consisted of an intracellular EG concentration of 6 mol/L and a final cell volume that was equal to the isotonic volume, cell shrinkage at the end of EG loading terminated at the isotonic cell volume, as expected. However, when the goal state was defined as 6 mol/L EG without specifying the final cell volume, shrinkage terminated at the minimum volume limit. Because of this additional shrinkage a relatively short EG loading phase was required to achieve the goal concentration. This shorter EG loading phase corresponded with a tenfold reduction in the toxicity cost (*J*_α_) associated with the CPA addition process, as shown in the bottom panel of Figure 1.

Figure 1 also compares two different approaches for parameterizing the solution composition for use in the optimization algorithm. In our previous study, we parameterized *m*_1_(*τ*) and *m*_2_(*τ*) using 49 equally spaced time segments with linear concentration changes in each segment [16]. Figure 1 compares this piecewise linear parameterization approach with two-step and three-step piecewise constant procedures, which are comparatively easy to implement experimentally. The piecewise constant procedures were optimized using a goal state of 6 mol/L intracellular EG, without specifying the final cell volume. Both the two-step and three-step piecewise constant procedures (green and blue lines, respectively) involved exposure to EG solutions lacking non-permeating solute in the first step, which caused the cells to shrink and then swell to the maximum volume limit. In addition, both procedures had a final step in which the cells were induced to shrink to the minimum volume limit by exposure to a hypertonic EG solution. These piecewise constant procedures were both shorter than the corresponding piecewise linear procedure with the same goal state (orange line). However, the piecewise constant procedures yielded a toxicity cost that was slightly larger than that obtained using the corresponding piecewise linear procedure. Table 1 summarizes the toxicity costs associated with each of the four different EG loading procedures described above.

**Table 1:** Comparison of mathematically optimized methods for equilibration of human oocytes with 6 mol/L EG

**Procedure**	**Concentration parameterization**	**Goal cell volume**	**Goal EG Conc. (*s***^**f**^**/*w***^**f**^**)**	**Toxicity (*J***_**α**_**)**
Addition	Piecewise linear	Isotonic	30	396
Addition	Piecewise linear	Not specified	30	32.3
Addition	2-step piecewise constant	Not specified	30	49.5
Addition	3-step piecewise constant	Not specified	30	42.7
Removal	Piecewise linear	Isotonic	0	38.4
Removal	Piecewise linear	Not specified	0	12.1
Removal	2-step piecewise constant	Not specified	0	12.8
Removal	3-step piecewise constant	Not specified	0	12.4

**Figure 2:**
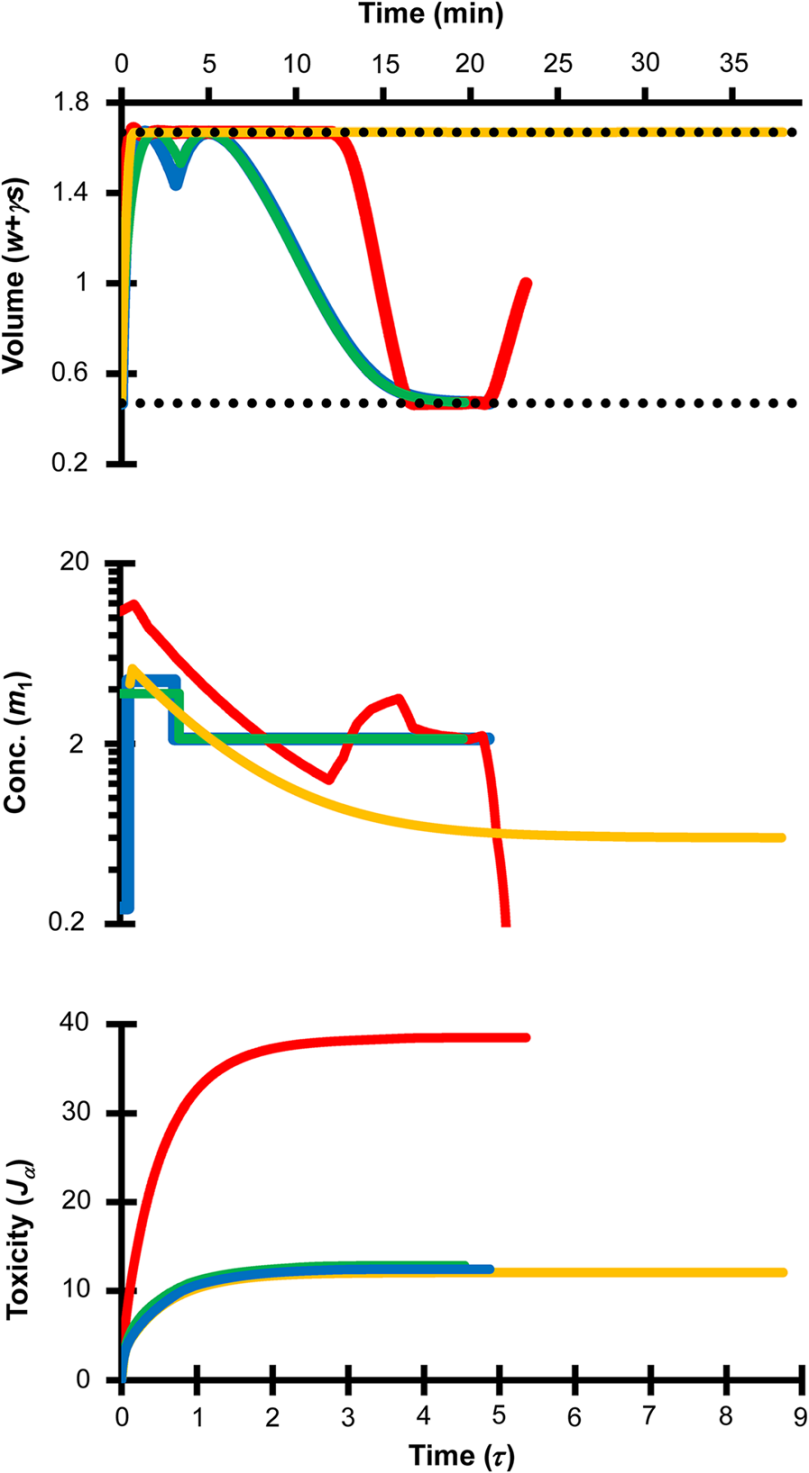
**Comparison of mathematically optimized protocols for removal of 6 mol/L EG from human oocytes.** The red line shows results from our previous study [16], which involved piecewise linear parameterization of *m*_1_(*τ*) and *m*_2_(*τ*) and a goal state fixed at the isotonic cell volume. The orange line shows results for the same piecewise linear parameterization of *m*_1_(*τ*) and *m*_2_(*τ*), but with a goal state that was not fixed at a specified final volume. The green and blue lines show two-step and three-step piecewise constant procedures, which had goal states that were not fixed at a specific final volume. The horizontal dotted lines in the top figure show the osmotic tolerance limits. Note that the nondimensional non-permeating solute concentration *m*_1_ can be converted to molal units by multiplying by 0.3 Osm/kg.

Figure 2 examines the effect of the mathematical optimization approach on procedures for removal of 6 mol/L EG from human oocytes. In all cases, the initial state for EG removal was assumed to be the corresponding final state after EG addition shown in Figure 1. All of the optimized EG removal procedures consisted of exposure to solutions containing non-permeating solutes, but lacking EG. In addition, all of the procedures resulted in swelling to the maximum volume limit. The red line shows the results of our previous study, which assumed a piecewise linear concentration profile and a goal state fixed at the isotonic cell volume. For comparison, the orange line shows the piecewise linear procedure that is obtained when the final cell volume is not fixed. As shown in the bottom panel of Figure 2, the predicted toxicity cost *J*_α_ was substantially higher in our previous study. This is primarily a result of differences in the cell volume before initiating the EG removal process. In our previous study, the cells were at their isotonic volume at the end of EG addition and hence started at the isotonic volume for EG removal. In contrast, the procedure designed without specifying the final cell volume started with the cell volume at the minimum volume limit. Consequently, swelling to the maximum volume limit resulted in greater dilution of the intracellular EG, leading to a lower toxicity cost. Two-step and three-step piecewise constant procedures are shown with green and blue lines, respectively. Both procedures were designed using a goal state that was not fixed at the isotonic cell volume. The toxicity cost associated with the two-step and three-step procedures was nearly identical to that obtained using the corresponding piecewise linear procedure, but much lower than the piecewise linear procedure with an isotonic final cell volume. In general, EG removal is predicted to be less toxic than EG addition, as can be seen by comparing the toxicity costs shown in Figures 1 and 2. These results are summarized in Table 1.

**Figure 3:**
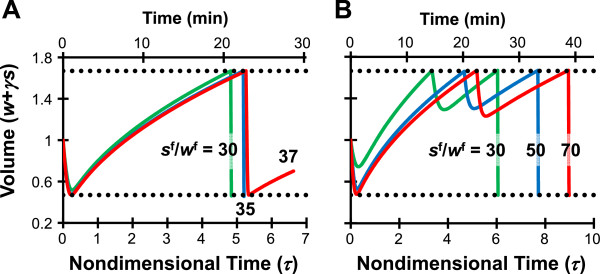
**Effect of increasing the goal concentration *s***^**f**^**/*w***^**f **^**on oocyte volume response during EG addition using two-step (A) and three-step (B) piecewise constant procedures.** The horizontal dotted lines show the osmotic tolerance limits. Goal concentration values *s*^f^/*w*^f^ = 30, 35, 37, 50 and 70 correspond with intracellular EG concentrations of 9, 10.5, 11.1, 15, and 21 Osm/kg (in molal units), or approximately 6, 6.6, 6.9, 8.2 and 9.7 mol/L (in molar units).

Although we nominally considered 6 mol/L EG to be a vitrifiable concentration to design the CPA addition and removal procedures shown in Figures 1 and 2, the actual concentration needed to vitrify depends on the cooling and warming rates. Therefore, in Figure 3 we examine the effect of increasing the goal state concentration on two-step and three-step piecewise constant EG addition procedures. In general, the final step of the EG addition procedure was short and consisted of rapid shrinkage to the minimum volume limit. However, as can be seen in Figure 3A, two-step procedures underwent a transition between goal state concentrations of 6.6 mol/L (*s*^f^/*w*^f^ = 35) and and 6.9 mol/L (*s*^f^/*w*^f^ = 37) in which the duration of the second step increased dramatically. This transition corresponded with the point at which the maximum amount of EG was loaded into the cells during the first step of the procedure. Maximum EG loading occurs when the cells are exposed to the EG concentration that causes shrinkage to the minimum volume limit and then equilibrated in this solution until the cell volume reaches the maximum volume limit. Beyond the transition point, maximal EG loading in the first step was not sufficient to allow the cells to achieve the goal EG concentration in the second step by shrinkage alone. Thus, further loading of EG had to be achieved by allowing the cells to partially equilibrate with a high EG concentration in the second step. For three-step procedures, this type of transition was not observed for goal concentrations up to 10.3 mol/L (*s*^f^/*w*^f^ = 80).

**Figure 4:**
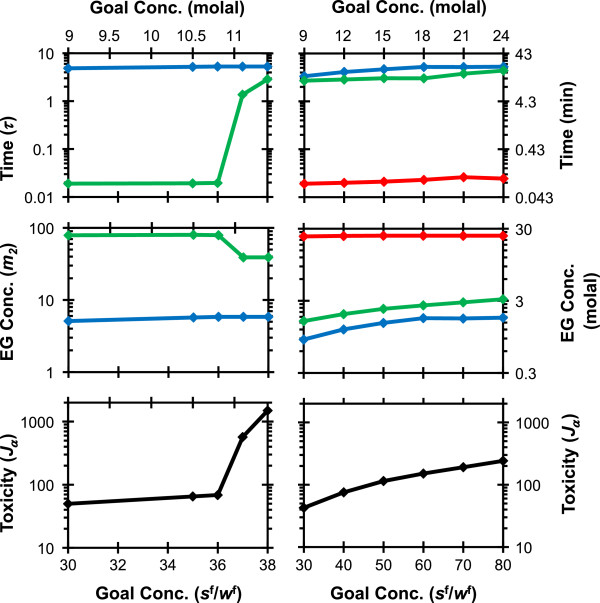
**Two-step (left) and three step (right) piecewise constant EG addition procedures as a function of the goal state concentration *s***^**f**^**/*w***^**f**^**.** The toxicity cost *J*_α_ at the end of the procedure, the EG concentration in each step of the procedure and the duration of each step are shown. Symbols show predicted values and the lines are provided to guide the eye. The colors blue, green and red represent steps 1, 2 and 3 respectively.

Figure 4 shows a more detailed comparison of the optimal procedures obtained for goal state concentrations ranging from *s*^f^/*w*^f^ = 30 to *s*^f^/*w*^f^ = 80. For two-step procedures, we can see that when the goal state is greater than *s*^f^/*w*^f^ = 36, the duration of the second step dramatically increases, leading to a substantial increase in the toxicity cost *J*_α_. For three step procedures, a similar abrupt increase in toxicity cost was not observed. In general, as the goal EG concentration increased, so did the predicted toxicity cost.

While the two-step and three-step procedures illustrated in Figures 1, 2, 3 and 4 are much easier to implement than the piecewise linear procedures, there are some practical issues that will need to be considered before such procedures are adopted clinically. Thus, to improve the optimized procedures, we examined the effects of including additional practical constraints in the optimization algorithm (see Table 2). The EG loading procedures presented above call for an extremely short final step. However, physical limits exist to how quickly the final addition step can be performed before cooling can be initiated. Therefore, we limited the step duration to the unitless equivalent of one minute in the optimization algorithm. In addition, the final step in the loading methods described above consists of exposure to a highly concentrated EG solution (i.e., *m*_2_ = 80, or about 10.3 mol/L), whereas it is more common to expose the cells to the minimum concentration necessary to achieve vitrification in the final step. Therefore, we constrained the EG concentration *m*_2_ using an upper limit equal to the goal concentration. Finally, the EG loading solutions described above only contain EG and water; the lack of ions and buffering salts in these loading solutions may cause damage that is not accounted for in the toxicity cost function. Therefore, we also imposed a constraint on the concentration of non-permeating solutes, limiting the concentration to at least 0.05 osmoles/kg (i.e., *m*_1_ > 0.167).

**Table 2:** Effects of parameter constraints on optimized piecewise constant procedures for equilibration of human oocytes with EG

**Procedure**	**Step**	**Non-permeating solute, *M***_**1 **_**(Osm/kg)**	**EG, *M***_**2 **_**(Osm/kg)**	**Time, *t *** **(min)**	**Toxicity (*J***_**α**_**)**
**Constraints:**		**0 ≤** ***M***_**1**_ **≤ 24**	**0 ≤** ***M***_**2**_ **≤ 24**	***t*** **≥ 0**	
Addition	1	0	1.4	20	130
	2	0	2.4	16	
	3	0	24	0.094	
Removal	1	1.8	0	3.6	22
	2	0.66	0	15	
**Constraints:**		**0 ≤** ***M***_**1**_ **≤ 24**	**0 ≤** ***M***_**2**_ **≤ 24**	***t*** **≥ 1**	
Addition	1	0	1.3	19	240
	2	0	2.4	16	
	3	0	17	1.0	
Removal	1	2.0	0	3.6	25
	2	0.65	0	15	
**Constraints:**		**0 ≤** ***M***_**1**_ **≤ 24**	**0 ≤** ***M***_**2**_ **≤ 16**	***t*** **≥ 1**	
Addition	1	0	1.4	20	250
	2	0	2.5	16	
	3	1.2	16	1.0	
Removal	1	1.8	0	3.5	22
	2	0.68	0	13	
**Constraints:**		**0.05 ≤** ***M***_**1**_ **≤ 24**	**0 ≤** ***M***_**2**_ **≤ 16**	***t*** **≥ 1**	
Addition*	1	0.050	1.4	24	
	2	0.050	2.4	20	280
	3	1.2	16	1.0	
Removal*	1	1.8	0	3.6	23
	2	0.66	0	14	

The goal state for EG addition was *s*^f^/*w*^f^ = 53.7, which is equivalent to an intracellular concentration of 8.5 mol/L, or 16 Osm/kg.

**Figure 5:**
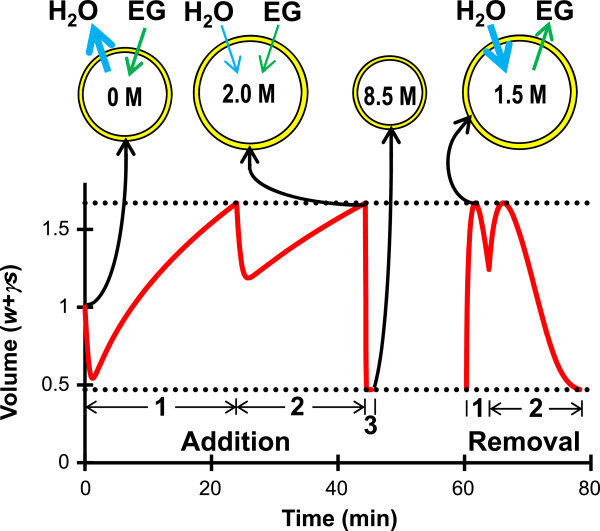
**Addition and removal of 8.5 mol/L EG using the methods indicated with asterisks in Table**2**.** The intracellular EG concentration (in mol/L) and transmembrane fluxes of water and EG are illustrated at several points in the CPA addition and removal process. During EG addition, the extracellular EG concentration was equal to 1.3 mol/L, 2.1 mol/L and 8.5 mol/L during steps 1, 2 and 3, respectively. The horizontal dotted lines show the osmotic tolerance limits.

*These procedures are illustrated in Figure 5.

Table 2 shows the effects of these practical constraints on procedures for addition and removal of EG. We designed procedures using a goal concentration of 8.5 mol/L because this EG concentration is expected to allow vitrification of the sample at the cooling and warming rates that are achievable using 1/4 mL freezing straws. When the step duration was limited to a minimum of 1 min, the only essential difference was an increase in the duration of the final addition step and a corresponding increase in the predicted toxicity cost by nearly two-fold. On the other hand, constraining the EG concentration to a maximum of 8.5 mol/L (i.e., *m*_2_ = 53.7) had very little effect on the toxicity cost. The main difference is that the resulting procedure calls for a non-zero concentration of non-permeating solute in the final addition step. Limiting the non-permeating solute concentration to a minimum of 0.05 Osm/kg resulted in longer equilibration times in steps one and two, and a corresponding modest (< 15%) increase in the toxicity cost. All of the parameter constraints considered in Table 2 resulted in nearly identical procedures for EG removal.

The procedures indicated with asterisks in Table 2 represent practical methods for equilibration of human oocytes with 8.5 mol/L EG. These EG addition and removal procedures are illustrated in Figure 5. The first two EG loading steps consist of cell shrinkage due to water efflux followed by swelling to the maximum osmotic tolerance limit as both water and EG enter the cell. At the end of the second step, the cell is predicted to reach an intracellular concentration of about 2 mol/L. In the third loading step, the cell rapidly shrinks due to water efflux and reaches an equilibrium volume at the lower osmotic tolerance limit; this serves to concentrate the intracellular EG to the goal concentration of 8.5 mol/L. The first step of EG removal involves exposure to a relatively hypotonic solution that causes water influx and concomitant swelling to the maximum osmotic tolerance limit. This swelling, coupled with efflux of EG, rapidly reduces the intracellular EG concentration to 1.5 mol/L. Together, these predictions show that by leveraging shrinking and swelling between the osmotic tolerance limits, addition and removal of 8.5 mol/L EG can be achieved while maintaining EG at low and relatively non-toxic concentrations throughout the majority of the process.

## Discussion

CPA induced cytotoxicity has been identified as a principal impediment to achieving successful vitrification [8]. However, the conventional approach for rational design of CPA equilibration procedures focuses only on avoidance of osmotic damage and does not consider mitigation of toxicity [9-11]. To address this deficiency, rational design approaches have recently been developed for minimizing protocol duration [12,13]; while these approaches would be expected to reduce toxicity compared with conventional methods, they do not account for the concentration dependence of toxicity. In our previous study [16] we described a new strategy for designing minimally-toxic CPA equilibration procedures using a concentration-dependent toxicity cost function. The resulting procedures are predicted to be less toxic than conventional methods for CPA equilibration as well as procedures with minimized duration. In this study we address two drawbacks of our previously reported mathematical optimization approach [16]. Our previous study relied on the concatenation of many linear changes in CPA and non-permeating solute concentrations which are difficult to achieve experimentally. Therefore, the primary objective of this study was to develop a method for designing multi-step CPA addition and removal procedures that are similar to conventional procedures with abrupt changes in CPA and non-permeating solute concentrations [7]. In addition, our previously reported optimization algorithm required cells to reach an isotonic final volume, potentially a suboptimal equilibration endpoint. Thus, an additional objective of this study was to evaluate alternate equilibration endpoints.

The two-step and three-step CPA equilibration procedures described in this study would be much easier to implement experimentally than the procedures described in our previous study [16]. Moreover, it is simpler and faster to predict optimal two-step and three-step procedures because there are fewer parameters to optimize and because an analytical solution to the membrane transport model is available for piecewise constant changes in solution composition [21]. However, it is important to evaluate the potential increase in toxicity associated with restricting the optimization to two-step and three-step piecewise constant concentration changes. Compared with the corresponding piecewise linear EG addition procedure, the two-step and three-step procedures had toxicity costs that were 50% and 30% higher, respectively (Table 1). Thus, it may be worthwhile to use continuous changes in concentration during CPA addition. However, CPA removal using two-step and three-step procedures is predicted to yield a toxicity cost that is nearly identical to that obtained using the corresponding piecewise linear CPA removal procedure, which indicates that the increased complexity of the piecewise linear procedure would probably not be worth the effort in this case. To fully evaluate the tradeoffs between experimental expediency and toxicity, it will be necessary to more precisely define the relationship between oocyte viability and the predicted toxicity cost.

The goal state defined in our previous study required that cells achieved an isotonic volume at the end of CPA addition. However, it is a common strategy to intentionally induce shrinkage in the final CPA addition step and to vitrify the sample while the cells are in the shrunken state [17,26-28]. For instance, multi-step vitrification procedures for oocytes commonly involve loading of CPA at relatively low concentrations followed by exposure to the final vitrification solution for a brief period of time directly before cooling [29-31]. In other words, with these procedures, the cooling process is initiated while the cells are in the shrunken state. The rationale behind this strategy is that water loss concentrates intracellular solutes, allowing a vitrifiable cytoplasm composition to be reached with a shorter exposure to the final vitrification solution [17]. Another advantage of vitrification in the shrunken state is that it facilitates removal of intracellular CPA after warming [17]. This is because the cell contains less total CPA in the shrunken state, and also has more capacity for swelling during the first removal step. The mathematically optimized procedures we describe in this study are consistent with this vitrification strategy in that the final CPA addition step comprises exposure to a concentrated CPA solution, which induces shrinkage to the minimum tolerable volume. Thus, our results provide a theoretical basis for the common practice of exposing cells to the final vitrification solution for a short time, and then initiating cooling while the cells are in the shrunken state.

The most unique aspect of our optimized procedures is that cells are loaded with CPA by inducing swelling to the maximum volume limit using a solution lacking non-permeating solutes (e.g., salts). In comparison, typical CPA loading solutions contain an isotonic concentration of non-permeating solutes and consequently do not induce swelling. Swelling is advantageous because it allows a given amount of CPA to be loaded into the cells using a relatively low CPA concentration. This is because the amount of intracellular CPA is equal to the product of the intracellular concentration and the cell volume. To our knowledge, loading CPA intracellularly while forcing cells to be in a swollen state is a novel result of our toxicity minimization strategy. While this approach is promising, it may be damaging to expose oocytes to solutions lacking salts because of potential perturbations in ion homeostasis. Studies with red blood cells show that complete lack of salts in the extracellular medium causes the cell membrane to become leaky, resulting in substantial loss of intracellular ions over a period of hours [32,33]. However, the presence of even a small amount of salt in the extracellular medium dramatically slows the rate of ion leakage [32,33]. This suggests that it may be possible avoid problems with ion leakage by including some minimal concentration of salts in the CPA loading solution. Recently, Karlsson and colleagues showed that mouse oocytes are not damaged by exposure to a CPA solution containing only 0.05 Osm/kg salts [34]. Therefore, we also optimized a CPA loading procedure using 0.05 Osm/kg as a minimum constraint on the non-permeating solute concentration (Table 2 and Figure 5). The resulting procedure still takes advantage of swelling, and hence would be expected to be much less toxic than conventional CPA loading methods, and is only marginally more toxic than our optimized protocols without the minimal salt constraint (Table 2).

Many studies are available describing the vitrification of human oocytes. In particular, the study by Kuwayama et al. [29] resulted in 7 healthy babies and 3 ongoing pregnancies at the time of publication. This study was also the first to use the Cryotop cooling device, a minimal volume device that offers an alternative to vitrification in freezing straws by taking advantage of the higher cooling and warming rates achieved with smaller sample volumes. The procedure for EG loading described by Kuwayama et al. results in a calculated toxicity of *J*_α_ = 60.6. Using the same goal state (an EG concentration of 5 mol/L, or *s*^f^*/w*^f^ = 23), our toxicity-minimization strategy predicts a procedure with a twofold lower toxicity of *J*_α_ = 29.3. It is important to note that the procedure described by Kuwayama et al. is predicted to yield oocyte volumes that exceed the osmotic tolerance limits that we used for designing our mathematically optimized method. Since the procedure reported by Kuwayama et al. has been successful, this may indicate that the osmotic tolerance limits that we used in this study were too restrictive. Broadening the osmotic tolerance limits would be expected to lead to even further reductions in the toxicity cost or increases in maximally achievable CPA concentration at the same cost.

While successful, the disadvantage of the Cryotop method employed by Kuwayama et al. [29] is that it is a potentially nonsterile system, where cells are directly exposed to liquid nitrogen. This open system is a requirement due to the ultrahigh cooling rates needed to avoid crystallization at such low CPA concentrations. However, if we assume that the calculated toxicity from their protocol, *J*_α_ = 60.6, is acceptable, then we can use our optimization approach to determine the maximal EG concentration that would result in the same level of toxicity. In this case we would be able to achieve a much increased goal concentration of approximately 6.6 mol/L (*s*^f^*/w*^f^ = 35) using two-step or three-step toxicity minimized procedures. This approach is useful because with higher goal concentrations, it is possible to achieve vitrification using less extreme cooling and warming rates. Thus, the ability to reach higher goal concentrations without significant cytotoxicity would enable the use of other devices that offer more sterility but have a greater thermal mass, such as freezing straws, and would offer considerably more margin for error in cooling and warming rates under the present Cryotop protocol.

Therefore, instead of minimizing toxicity under current cooling regimes such as the Cryotop method, we may use our optimization approach to calculate the anticipated added cost of achieving a concentration that would facilitate vitrification under more sterile conditions. In particular, Baudot and Odagescu [35] determined that a 50% w/w EG solution required a cooling rate of 11°C/min to achieve vitrification and a warming rate of 853°C/min to prevent devitrification. Cooling rates up to 2000°C/min can be achieved by directly immersing 1/4 mL freezing straws into liquid nitrogen, and warming rates up to 3000°C/min can be achieved by immersing straws into a 25°C water bath [36]. Thus, 50% w/w EG should conservatively enable vitrification at the cooling and warming rates achievable using freezing straws. An EG concentration of 50% w/w corresponds with a goal state of *s*^f^*/w*^f^ = 53.7. Using our toxicity-minimized procedures, achieving a goal state of *s*^f^*/w*^f^ = 53.7 would result in a toxicity of *J*_α_ = 130 (Table 2). This is larger than the predicted toxicity cost associated with the procedure reported by Kuwayama et al. [29] which has been proven successful. However, greater toxic damage may be an acceptable tradeoff for increased sterility and improved stability of the glassy state during storage. The clinical application of this approach will require a more precise understanding of the cost function *J*_α_, and the determination of acceptable values of this cost in the context of reproductive medicine.

Our results show that to minimize toxicity during CPA addition, the final step should induce shrinkage to the minimum volume limit and last only long enough for this minimum volume to be achieved. For instance, the two-step and three-step CPA addition procedures shown in Figure 1 had final steps with durations of about 5 seconds. However, it may not be practical to perform a 5 second equilibration with sufficient accuracy and repeatability for clinical application. A distinct advantage of our approach is that it allows the determination of optimal protocols even after the addition of practical design constraints to the problem. Most previously reported CPA equilibration procedures for vitrification of human oocytes involve exposure to the final vitrification solution for at least 30 seconds [29-31]. Thus, we assumed that a one-minute final step would be feasible and determined optimal two-step and three-step procedures with this constraint (see Table 2). Interestingly, when such procedures were designed using a maximum concentration constraint equal to the goal concentration, the final addition step called for the presence of non-permeating solute at a concentration of approximately 1 Osm/kg. This is consistent with the common practice of including 0.5-1 mol/L sucrose in the final vitrification solution for human oocytes [29-31]. The presence of non-permeating solute in the final vitrification solution is potentially advantageous because it results in equilibration of the cells in a shrunken state. For example, exposure to the final solution compositions shown in Table 2 is predicted to cause rapid shrinkage and subsequent equilibration at the minimum volume limit in less than 20 seconds, as shown in Figure 5. These procedures would be expected to be relatively robust to variations in the exposure time in the final CPA addition step, since equilibrium is achieved quickly.

The optimized procedures for EG removal presented here call for exposure to solutions containing non-permeating solutes, but lacking EG. However, some residual EG would be present in practice, regardless of the method for changing the extracellular composition. To examine the potential effects of residual EG, minimum constraints can be imposed on the EG concentration during each removal step. If the EG concentration is constrained to a 20-fold dilution in each step, the toxicity cost associated with the resulting procedure is about 40% higher than that obtained when the EG concentration is zero in each step. A 100-fold dilution in each step is only associated with a 6% increase in toxicity cost. Overall, these increases in toxicity would not be expected to substantially effect of the outcome of the cryopreservation process, since EG removal is still be predicted to be much less toxic than EG addition.

Although we used our optimization algorithm for human oocytes in this study, our approach is applicable to any cell type given the necessary biophysical parameters (i.e., the membrane permeability values and osmotic tolerance limits). Moreover, our general approach of minimizing a toxicity cost function provides a framework for optimizing other important aspects of the CPA equilibration process. For instance, it is generally recognized that CPA toxicity is reduced at lower temperatures (e.g., 4°C), but CPA loading also takes longer at low temperatures because the cell membrane permeability is lower. Thus, selection of the optimal temperature for CPA loading is not trivial and arguments have been presented for CPA equilibration at both low temperatures [27] and high temperatures [37]. Our optimization approach also provides a framework for rational comparison of different CPA types in terms of their toxicity. To extend our approach to optimization of factors such as temperature and CPA type will require an improved understanding of the effect of these factors on the rate of damage due to toxicity, and formulation of a toxicity cost function that accounts for these factors.

Another advantage of our approach is that the toxicity cost function provides a quantitative indicator of cell damage after cryopreservation, facilitating rational evaluation of feasibility. If the expected cost under the optimal protocol is unacceptable, exceeding a limit that indicates a significant level of damage, a completely new approach must be tried that mitigates this cost. For example, it may be possible to reduce toxicity by using a different combination of CPAs or by carrying out the procedure at a different temperature. Importantly, the model results can be used to direct the research focus to the source of damage. This aspect is unique to our approach and has the potential to save time by identifying non-feasible approaches without the need for fruitless experiments. To realize these benefits, it will be necessary to clarify the factors affecting the toxicity cost function, as well as the relationship between the cost function and cell viability for the cell type of interest.

## Conclusions

In this study we have presented an adaptation of our toxicity-minimization strategy for predicting CPA addition and removal procedures. In particular, we have modified our previous strategy which relied on continuous concentration changes and instead predict procedures based on piecewise constant concentration changes. These new procedures are not only similar to conventional procedures but are also much simpler to implement experimentally. The mathematical algorithm is based on the minimization of a toxicity cost function, which describes the effect of CPA concentration on cytotoxicity. Although these procedures still require experimental validation, we have provided theoretical evidence suggesting that our procedures would reduce toxic damage relative to procedures that are currently in use. The employment of this cost function allows for rational comparison of potential experimental designs and facilitates the generation of cell damage hypotheses in the context of cryopreservation protocols. Finally, our strategy also provides a structure for incorporating other factors into the model-based design of toxicity-minimized vitrification procedures, including the effects of temperature on CPA toxicity.

## Appendix

An analytical solution has previously been published for the two-parameter membrane transport model [21], but not explicitly for the nondimensional form of transport model given in system (1). Parameterizing the equation in the time variable using Eq. 7 is equivalent to multiplying the right hand side of each equation by *w* and yields the linear system

(A1) $$\begin{array}{l} \frac{\mathrm{dw}}{\mathrm{dx}}=-\left(m_{1}+m_{2}\right)w+s+1,\\ \frac{\mathrm{ds}}{\mathrm{dx}}=bm_{2}w-\mathrm{bs}, \end{array}$$

which retains the same initial conditions as system (1). This system may be solved using standard techniques. The analytical solution in terms of the nondimensional variables *w* and *s* is, with m_1_ ≠ 0

(A2) $$w=\frac{1}{m_{1}}+\frac{m_{1}w^{i}-1-C_{1}}{2m_{1}}\exp\left(r_{1}x\right)+\frac{m_{1}w^{i}-1+C_{1}}{2m_{1}}\exp\left(r_{2}x\right),$$

(A3) $$s=\frac{m_{2}}{m_{1}}+\frac{m_{1}s^{i}-m_{2}+C_{2}}{2m_{1}}\exp\left(r_{1}x\right)+\frac{m_{1}s^{i}-m_{2}-C_{2}}{2m_{1}}\exp\left(r_{2}x\right),$$

where *w*^i^ and *s*^i^ are the initial values of *w* and *s*, respectively, and the constants *C*_1_, *C*_2_, *r*_1_ and *r*_2_ are defined as

(A4) $$C_{1}=\frac{m_{1}-m_{2}+2m_{1}s^{i}-m_{1}w^{i}\left(m_{1}+m_{2}\right)+b\left(m_{1}w^{i}-1\right)}{\sqrt{{\left(b+m_{1}+m_{2}\right)}^{2}-4bm_{1}}},$$

(A5) $$C_{2}=\frac{\left(m_{1}+m_{2}\right)\left(m_{2}-m_{1}s^{i}\right)+b\left(m_{2}+m_{1}s^{i}-2m_{1}m_{2}w^{i}\right)}{\sqrt{{\left(b+m_{1}+m_{2}\right)}^{2}-4bm_{1}}},$$

(A6) $$r_{1}=-0.5\left(b+m_{1}+m_{2}+\sqrt{{\left(b+m_{1}+m_{2}\right)}^{2}-4bm_{1}}\right),$$

(A7) $$r_{2}=-0.5\left(b+m_{1}+m_{2}-\sqrt{{\left(b+m_{1}+m_{2}\right)}^{2}-4bm_{1}}\right).$$

This analytical solution was used in our optimization algorithm to predict changes in cell volume and intracellular CPA concentration, thus allowing evaluation of the cell volume constraints as well as the toxicity cost function. We restricted *m*_1_ > 10^− 4^.

Note that this solution is in the new time space. To recover the original nondimensional time *τ* from the time-like variable *x* we must integrate Eq. A2:

(A8) $$\tau =\int_{0}^{x}w\left(x\right)\mathrm{dx}.$$

## Competing interests

The authors declare that they have no competing interests.

## Authors’ contributions

AD developed and tested the numerical method and prepared the initial draft of the manuscript under the supervision of AH. JB participated in analysis of results, modeling and optimization and manuscript preparation. All authors read and approved the final manuscript.
